# Single-Stage Reverse High Tibial Osteotomy and Total Knee Arthroplasty for Valgus Extra-Articular Deformity After Failed HTO: A Matched-Pair Pilot Study †

**DOI:** 10.3390/jcm15124700

**Published:** 2026-06-17

**Authors:** Maximilian Jörgens, Wolfgang Reng, Julian Karpf, Edna De la Ossa Cordoba, Steffen Klingbeil, Rolf Schipp, Johannes Becker

**Affiliations:** 1 endogap, Joint Replacement Institute, Klinikum Garmisch-Partenkirchen, 82467 Garmisch-Partenkirchen, Germany; 2 Department of Orthopaedics and Trauma Surgery, Musculoskeletal University Center Munich (MUM), University Hospital, LMU, 80336 Munich, Germany; 3 Department of Trauma Surgery, BG Unfallklinik Murnau, 82418 Murnau, Germany; 4 Institute for Biomechanics, Paracelsus Medical University, 5020 Salzburg, Austria

**Keywords:** total knee arthroplasty, high tibial osteotomies, arthroplasty, osteoarthritis, functional outcome score, reversed HTO

## Abstract

**Background**: High tibial osteotomy (HTO) is an established joint-preserving treatment for medial knee osteoarthritis, but total knee arthroplasty (TKA) may be required if the disease progresses. This pilot study evaluates the radiological and functional outcomes of simultaneous limb axis correction using reverse HTO (rHTO) during TKA compared to a primary TKA control group without previous HTO. **Methods**: In this retrospective matched-pair study, nine patients with previous valgus HTO underwent varus correction via rHTO combined with TKA using cruciate-retaining implants. Outcomes were compared to a primary TKA control group. Radiographic parameters (MPTA, mLDFA, aHKA, JLO) and clinical scores (Oxford Knee Score, LEFS, KOOS, TAS, FIPS) were assessed pre- and postoperatively. Statistical significance was set at *p* < 0.05. **Results**: Preoperative alignment differed significantly between groups (MPTA, aHKA, JLO; all *p* ≤ 0.001). Postoperatively, both cohorts achieved neutral mechanical alignment showing no statistically significant differences in descriptive parameters. CPAK classification showed convergence to neutral alignment types. Functionally, mean postoperative clinical scores, including KOOS subdomains, LEFS, and Oxford Knee Score, were numerically close between groups, though confidence intervals were wide due to the limited sample size (all *p* > 0.05). **Conclusions**: This pilot study demonstrates the technical feasibility of single-stage rHTO and TKA, showing encouraging descriptive clinical and radiographic profiles in this small, highly specific cohort. Given the exploratory nature of this study, larger trials are required to definitively evaluate potential clinical differences.

## 1. Introduction

High tibial osteotomy (HTO) is an established, joint-preserving procedure used to treat unilateral, medial compartment knee osteoarthritis, especially in younger, active patients with a varus leg axis [1,2]. The biomechanical principle of HTO is to shift the mechanical weight-bearing axis of the leg from the diseased medial compartment to the healthier lateral compartment, which is typically achieved by creating a slight valgus correction [2,3]. The medial open wedge osteotomy (owHTO) has proven superior to the lateral closed wedge osteotomy (cwHTO) in terms of long-term results [4]. The latter more frequently requires revision with the implantation of a bicondylar knee prosthesis (total knee arthroplasty, TKA) [4].

However, a critical factor for the failure of an HTO is iatrogenic valgus overcorrection [5]. This pathological change can be precisely described using the CPAK (Coronal Plane Alignment of the Knee) classification, which categorizes knee phenotypes based on constitutional alignment (arithmetic Hip–Knee–Ankle angle, aHKA) and joint line obliquity (JLO) [6]. In the healthy population, varus and neutral phenotypes such as CPAK types I, II, IV, and V are predominantly found, while valgus phenotypes such as CPAK type III, VI, and especially IX are rare and considered non-physiological [7]. Studies show that a medial opening HTO often shifts patients from a common CPAK type I (constitutional varus with apex-distal JLO) to a rare, pathological CPAK type VI or IX [8]. Such a shift could represent a deviation from the phenotype of natural alignment according to Bellemans et al. [9]. In case of a failed HTO with valgus overcorrection, surgery involves a “reverse” HTO (rHTO), typically in the form of a medial closing wedge osteotomy. The CPAK classification has proven to be clinically relevant in TKA for both preoperative planning and postoperative phenotype assessment, as valgus malalignment after TKA is associated with inferior functional outcomes and higher revision rates [6,10,11,12,13]. Compared to patients undergoing primary TKA, those with a previous HTO show significantly worse functional outcomes, as reflected by higher pre- and postoperative pain levels, longer operating times, and a more frequent use of modular implants [14,15]. To adequately address the underlying extra-articular deformity (EAD) and optimize biomechanics, combined one- or two-stage strategies (rHTO and TKA) have been developed, with treatment selection primarily guided by deformity complexity and patient-specific factors (e.g., age, physiological status, and soft tissue conditions) [8,16,17,18,19,20].

This procedure can be understood as a form of phenotypic restoration: the pathological CPAK configuration (e.g., type IX) is actively restored to a more neutral and biomechanically stable phenotype (e.g., type I, II, IV, V) [6]. This approach aligns with modern concepts of individualized alignment, which recognize that a significant portion of the population has a “constitutional varus” [6,9,12].

Despite the strong biomechanical rationale for performing combined rHTO+TKA in patients with failed HTO and valgus EAD, there is a notable lack of high-quality comparative studies in the literature. Most existing reports are limited to small case series or analyses that group different types of EAD together, without specifically isolating this particularly challenging patient population. Consequently, it remains unclear whether this more complex combined procedure can achieve radiological and functional outcomes comparable to those of a standard primary TKA.

The aim of the present study was therefore to perform a matched-pair analysis comparing radiological and functional outcomes of patients undergoing single-stage rHTO+TKA with those of a control group receiving primary TKA. Due to the pilot nature of this investigation, the study aimed to descriptively explore whether the intervention group achieves satisfactory postoperative alignment and functional clinical scores at follow-up. Such findings would support the concept that simultaneous correction of the EAD effectively mitigates the disadvantages typically associated with performing TKA after a prior HTO.

## 2. Materials and Methods

### 2.1. Study Design and Population

This retrospective matched case–control study comprised nine patients who underwent single-stage rHTO and TKA procedures between 2013 and 2023 at a single institution. Each patient was individually matched to a control subject who received TKA alone, based on primary indication (osteoarthritis), age at surgery (±5 years), gender, body mass index (BMI ±3), American Society of Anesthesiologists (ASA) classification, and date of surgery (±3 months). To ensure a homogeneous study population and eliminate confounding factors, strict selection criteria were applied.

For the intervention group, inclusion criteria required a history of a failed, valgus-producing HTO with secondary osteoarthritis. Within this group, extra-articular deformity (EAD) was defined as deviation from the physiological mechanical limb axis, including valgus alignment, joint line obliquity (JLO) towards the lateral side, and a medial proximal tibial angle (MPTA) ≥ 90°. Exclusion criteria for both the intervention and control groups were defined as active or recent joint infection (periprosthetic or superficial), neuromuscular disorders severely compromising lower limb function, and prior major knee fractures or tumor-related surgeries. Patients presenting with ligamentous instability or a history of prior arthroplasty were likewise excluded from consideration. To provide a comprehensive profile of the study cohort, additional variables such as smoking status, hemoglobin (Hb) levels pre- and postoperatively, and operative time were documented.

All participants were followed for a minimum of one year postoperatively. Preoperative status was assessed retrospectively for the Tegner Activity Score (TAS), while all other functional scores and radiographs were prospectively collected at a single timepoint during the final follow-up appointment. All intraoperative and postoperative complications, including revision surgeries, were recorded.

Ethical approval was granted by the local institutional review board (approval number 2024-1018), and all patients provided written informed consent.

### 2.2. Surgical Technique and Postoperative Care

**Figure 1:**
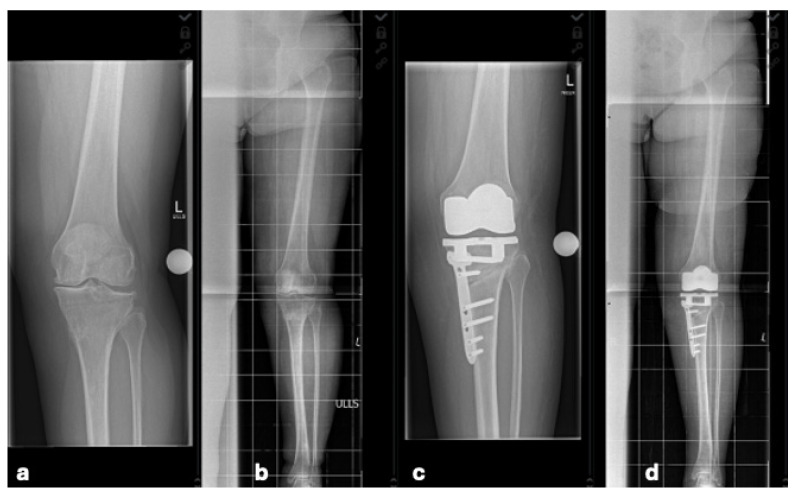
Preoperative and postoperative radiological assessment of a left knee undergoing a single-stage rHTO+TKA. (**a**) Preoperative anteroposterior knee radiograph and (**b**) full length standing radiograph showing secondary osteoarthritis and tibial extra-articular valgus deformity. (**c**) Postoperative anteroposterior knee radiograph and (**d**) full length standing radiograph demonstrating neutral alignment restoration after medial closed wedge rHTO with a locked plate and TKA. (rHTO: reversed high tibial osteotomy; TKA: total knee arthroplasty).

A conventional HTO typically corrects varus deformities by shifting the mechanical axis toward valgus. In contrast, the rHTO utilized in this study is a varus-producing procedure. The surgical rationale for combining rHTO with TKA targets patients with severe extra-articular valgus deformities, which frequently result from overcorrected prior open wedge HTO. Performing an isolated TKA in these complex cases requires massive asymmetric intra-articular bone resections and extensive ligament releases, compromising joint stability. Addressing the EAD directly at its apex restores a neutral mechanical axis and a physiological joint line. Crucially, this approach allows conservative intra-articular bone cuts and explicitly avoids extensive soft tissue releases. All operations were performed by a senior surgeon (WR). The single-stage procedure combined total knee arthroplasty and a simultaneous medial closed wedge rHTO. Regardless of the intraoperative sequence, the concurrent osteotomy successfully managed the EAD in all patients, allowing the exclusive utilization of standard cruciate-retaining (CR) implants and eliminating the need for constrained prostheses. Stable internal fixation was achieved using a locked anatomical plate system. Postoperative radiographs routinely confirmed stable implant fixation, accurate osteotomy closure, and successful restoration of the mechanical joint line (Figure 1).

Following surgery, patients followed a standardized rehabilitation protocol. Partial weight bearing limited to 20 kg was maintained for the first six weeks, followed by progressive weight bearing as tolerated. Physiotherapy was initiated postoperatively, supported by the routine use of a continuous passive motion device to facilitate early range of motion and functional recovery.

### 2.3. Radiologic Evaluation

Postoperative radiographs including anteroposterior (AP), lateral, and AP full-length weight-bearing views were analyzed for implant alignment, signs of migration or loosening, and evidence of osseous consolidation at the osteotomy site. Radiographic evaluation also included measurement of the medial proximal tibial angle (MPTA) and the mechanical lateral distal femoral angle (mLDFA) in full-length weight-bearing X-ray. From these values, the arithmetic hip–knee–ankle angle (aHKA = MPTA − mLDFA) and the joint line obliquity (JLO = MPTA + mLDFA) were calculated both preoperatively and postoperatively at follow-up. In one exceptional case, the final radiograph was obtained at a routine visit (minimum of 12 months postoperatively) rather than on the exact day of clinical scoring due to logistical reasons. Measurement of these angles was performed by one observer (MJ).

Based on the radiographic measurements, each leg was assigned a corresponding knee phenotype according to the CPAK classification (nine types), both before and after surgery [6]. In this classification, negative aHKA values indicate varus alignment, and positive values indicate valgus alignment [6]. CPAK sectors were defined using thresholds of ±2° for aHKA and 177°/183° for JLO.

### 2.4. Functional Evaluation

Functional scores such as the Lower Extremity Functional Score (LEFS), Tegner Activity Score (TAS), Oxford Knee Score (OKS), Knee Osteoarthritis Outcome Score (KOOS; including subscales: Pain, Symptoms, ADL (Activities of Daily Living), Sports (Sports and Recreation), and QoL (Quality of Life)) and Freiburg Index for Patient Satisfaction (FIPS) were reported during follow-up. Preoperative TAS values were retrospective. Prespecified MCID (minimal clinically important differences) thresholds were derived from previously published MCID and from previous studies defined as follows [21,22,23]: LEFS (±9 points), OKS (±5), TAS (±0.9), KOOS Pain (±9), KOOS Symptoms (±10.8), KOOS ADL (±10), KOOS Sports (±17.8), KOOS QoL (±12.7), and FIPS (±1). For the FIPS, since established MCID thresholds are not available in the current literature, a margin of ±1 point was determined in advance based on clinical expertise. Higher FIPS scores demonstrated better function.

### 2.5. Statistical Analysis

Data analyses were conducted using Python (Version 3.11, Python Software Foundation, Wilmington, DE, USA), IBM SPSS Statistics (Version 29, IBM Corp., Armonk, NY, USA) and jamovi (Version 2.6.23.0; The jamovi project, 2024). Measurement data were expressed as means and standard deviations (SD) for continuous variables, and as frequencies and percentages for categorical variables.

Normality was assessed using the Shapiro–Wilk test. Depending on distribution, paired *t*-tests or Wilcoxon signed-rank tests were used for continuous variables; categorical variables were compared with appropriate non-parametric tests. ICC was calculated with a two-way random effects model (absolute agreement). To prevent recall bias, intraobserver measurements were repeated after an interval of six months. ICC values were interpreted per Koo and Li (<0.50 poor, 0.50–0.75 moderate, 0.75–0.90 good, >0.90 excellent) [24].

To evaluate clinical outcomes relative to established MCID, 90% confidence intervals (90% CI) for the mean differences (Δ) between groups were calculated. Due to the pilot design of this study, a formal equivalence trial was not feasible. Instead, the overlap of these confidence intervals with the MCID margins was analyzed descriptively to reveal statistical power limitations.

All analyses were conducted in accordance with the matched-pair design of the study. Visualizations were generated using the Matplotlib library (https://matplotlib.org, accessed on 1 June 2025; Hunter JD).

## 3. Results

### 3.1. Study Population, Clinical Characteristics and Complications

A total of 18 patients (nine pairs) were included in the study. The median follow-up period was 36.4 months (IQR (interquartile range), 23.6 to 65.5 months). Table 1 presents the demographic and surgery-related characteristics of the cohort. Due to the matched-pair design, there were no significant differences between the groups in terms of age, sex, BMI (body mass index), ASA classification and smoker status. There were also no significant differences in pre- and postoperative hemoglobin (Hb) levels (*p* > 0.05).

In one case, anterior subsidence of the tibial plateau was noted during the first postoperative year. After radiographic confirmation of complete consolidation at the osteotomy cut, a tibial revision was performed. An unlinked cemented tibial revision component was implanted, and the associated bone defect was managed with autologous bone grafting. The postoperative course remained uneventful, with no further complications observed. The initial postoperative radiograph was used for radiological measurements to enable comparison of the primary realignment by rHTOs.

### 3.2. Radiologic Alignment Parameters

Pre- and postoperative radiological parameters were compared between the matched paired rHTO+TKA group and primary TKA group (Table 2). Reliability was consistently high across all parameters. Intraobserver agreement was excellent preoperatively (ICC: 0.95–0.95) and good to excellent postoperatively (ICC: 0.81–0.93). Interobserver agreement showed excellent preoperative (ICC: 0.912–0.93) and good postoperative reliability (ICC: 0.79–0.81). The mean absolute difference between measurements was <2° for all angles.

Preoperative alignment differed markedly between the two treatment groups. Patients undergoing rHTO+TKA presented with a mean preoperative aHKA of 6.4° valgus (±3.3), whereas those treated with isolated TKA had a mean preoperative aHKA of −1.9° varus (±3.9). Preoperative MPTA, aHKA, and JLO differed significantly (all *p* ≤ 0.001), reflecting distinct baseline alignments.

Postoperatively, both cohorts achieved alignment values within the range of mechanical neutrality. The rHTO+TKA group reached a mean postoperative aHKA of −0.2° varus (±2.9), while the TKA group showed a mean of −2.9° varus (±2.8). No statistically significant differences were observed in postoperative alignment parameters (all *p* > 0.05), suggesting that both procedures resulted in final limb alignment and joint line orientation without statistically significant differences.

**Figure 2:**
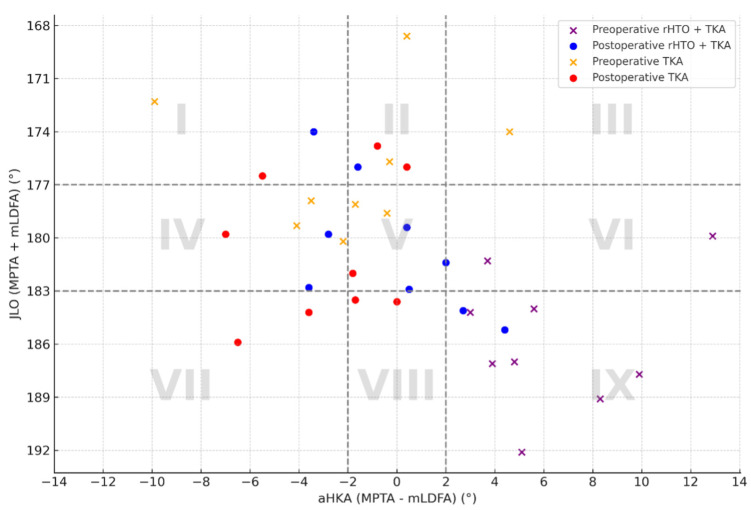
CPAK-based distribution of pre- and postoperative alignment across treatment groups. Each CPAK type (I–IX) is represented by a labeled sector, delineated by aHKA thresholds of ±2° and JLO thresholds of 177° and 183°. MPTA: medial proximal tibial angle; mLDFA: mechanical lateral distal femoral angle; aHKA: arithmetic hip–knee–ankle angle; JLO: joint line obliquity; rHTO: reversed high tibial osteotomy; TKA: total knee arthroplasty.

The distribution across CPAK types also varied between groups (Figure 2). Preoperatively, patients in the rHTO+TKA group were primarily categorized as CPAK Types IX (valgus alignment with distal apex lateral joint line; *n* = 7 (77.8%)) and VI (valgus alignment with neutral apex lateral joint line; *n* = 2 (22.2%)). Conversely, the isolated TKA group demonstrated a broader and more physiological distribution, with patients categorized as CPAK Type V (*n* = 2 (22.2%)), Type IV (*n* = 3 (33.3%)), Type III (*n* = 2 (22.2%)), Type II (*n* = 1 (11.1%)) and Type I (*n* = 1 (11.1%)). Following surgery, both groups demonstrated a convergence of CPAK types reflecting successful realignment toward mechanical neutrality and consistent joint line correction.

### 3.3. Functional Scores

Across all evaluated functional outcome measures—including the Lower Extremity Functional Scale (LEFS), Oxford Knee Score (OKS), KOOS subscales, Tegner Activity Score (TAS), and FIPS—no statistically significant differences were found between groups (*p* > 0.05 for all comparisons; Table 3).

**Figure 3:**
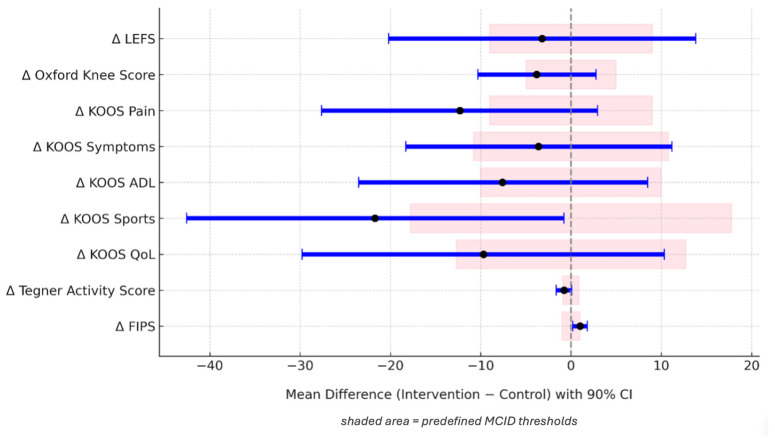
Mean differences with 90% confidence intervals (CIs) in functional outcomes comparing rHTO+TKA patients to TKA. Shaded areas represent predefined MCID thresholds for each score (*n* = 9 pairs); MCID: minimal clinically important difference.

In addition, none of the calculated 90% confidence intervals lay entirely within the predefined MCID thresholds, descriptively illustrating that this pilot study is underpowered to formally establish clinical equivalence (Figure 3). The KOOS Sports subscale showed numerical trends favoring the control group (mean-Δ = −21.7, *p* = 0.090), while the FIPS scores were numerically higher in the rHTO+TKA group (mean-Δ = +1.0, *p* = 0.053). No significant differences were found in any of the scores.

## 4. Discussion

This matched-pair study found that single-stage TKA with simultaneous rHTO in patients with failed HTO and valgus malalignment resulted in radiological and functional outcomes that did not differ significantly from standard primary TKA in this small cohort. At a minimum one-year follow-up, no significant differences were observed in postoperative alignment parameters (MPTA, aHKA, JLO) or in patient-reported outcomes (TAS, OKS, LEFS, KOOS, FIPS; all *p* > 0.05).

Despite greater anatomical deformity and surgical complexity, the intervention group showed outcomes that did not differ significantly, with mean preoperative valgus aHKA of 6.4° corrected to −0.2° varus. The control group shifted from −1.9° to −2.9° varus. These findings suggest that combined rHTO+TKA has the potential to restore a neutral mechanical axis and yield functional results without statistically significant differences compared to primary TKA, despite the challenges of prior HTO.

Residual EAD after a valgus-producing HTO (MPTA ≥ 90°) prevents neutral mechanical alignment, accelerates polyethylene wear and aseptic loosening, and compromises long-term function [15,25]. Correcting this deformity only with asymmetric bone cuts and extensive soft tissue releases can destabilize the collateral ligaments, often requiring constrained implants with poorer kinematics and durability. Similarly, Mader et al. highlighted that TKA following HTO is associated with higher complication rates and technical challenges, necessitating careful preoperative planning [15]. Song et al. demonstrated that conversion to TKA via re-osteotomy offers distinct surgical advantages, including reduced tibial bone resection, restoration of joint line height, and improved ligamentous balancing [26]. The strategy articulated in the previous study has the potential to minimize the risk of valgus alignment after TKA and poor clinical outcome [11,27].

Performing single-stage rHTO and TKA successfully normalizes tibial alignment, streamlines soft tissue balancing, and avoids the mechanical pitfalls of intra-articular overcorrection. Although the combined procedure increases operative time (117.0 min vs. 64.9 min), it consistently allows for symmetric bone resections and the exclusive use of standard CR implants. Based on the operative experience gained from this cohort, the authors specifically recommend implanting the TKA components with ligament balancing first, followed by the execution and fixation of the rHTO. This sequence provides a stable intra-articular baseline and ensures predictable soft tissue tensioning before the extra-articular alignment is definitively altered by the osteotomy, thereby successfully eliminating the need for more constrained implants. In their 2002 study, Radke and Radke showed good valgus or varus axis correction without navigation and good clinical results [19]. In a case report by Kubo et al. good clinical outcome was observed after navigation-assisted one-stage TKA with EAD and corrective femoral osteotomy [28]. So, the combination of rHTO and TKA is a valid alternative to pure soft tissue release techniques, particularly in young, active patients who initially underwent HTO. It enables the reconstruction of the limb axis in a correct alignment while preserving ligamentous structures [6,11,12]. Single-stage rHTO simplifies complex deformities into a more standard anatomy. This may allow for clinical and radiographic results not significantly different from those of primary TKA in non-deformed knees.

Postoperatively, no significant differences in MPTA, aHKA, or JLO were observed between the intervention and control groups, underscoring the precision of rHTO. The CPAK classification also demonstrated a clear harmonization of the preoperative valgus alignment toward neutral postoperative phenotypes, consistent with the findings of Corban et al. and Pujol et al., who highlighted its utility for both surgical planning and outcome assessment [6,7,12]. The lack of major differences in postoperative alignment between both groups supports the technical feasibility of the combined approach, even in the context of complex initial deformities. These results corroborate earlier investigations that emphasized the importance of axis correction for EAD before TKA implantation [8,16,17,18]. Catonné et al. reported similar success in a heterogeneous cohort of valgus and varus EAD patients treated with reconstructive rHTO and TKA, while Madelaine et al. demonstrated significant postoperative realignment following owHTO and TKA in pronounced varus deformities [8,18]. The present study builds on this evidence, demonstrating in a more narrowly defined, matched cohort that meticulous restoration of proximal tibial anatomy by correcting MPTA from 96.1° to 90.2° and JLO from 185.8° to 180.6° provides a stable, neutral platform for a well-balanced TKA with minimal need for soft tissue releases. This approach aligns with modern alignment concepts that aim to restore the pre-arthritic joint line for better soft tissue balance and biomechanics. Precise anatomical and phenotypic restoration likely explains the excellent functional outcomes and underscores the value of CPAK classification as a contemporary tool for planning and evaluating complex corrections.

A key strength of this study is that the functional outcomes of the rHTO+TKA cohort reflect not only relative success compared with the control group but also absolute clinical success against established benchmarks. The mean postoperative OKS (39.3 ± 10.9) exceeded the PASS threshold of ≥30 reported by Ingelsrud et al., indicating patient-perceived success [29]. This places the average outcome in the range of good clinical results, with only mild-to-moderate residual symptoms. KOOS results further support these findings, with mean scores of 81.2 ± 26.0 for Pain and 86.4 ± 22.4 for ADL, indicating substantial pain relief and functional recovery, consistent with the outcomes and PASS thresholds reported by Connelly et al. [30]. Due to the pilot character and small sample size of nine matched pairs, the resulting wide confidence intervals did not fall entirely within the predefined MCID thresholds. Notably, the KOOS Sports subscale demonstrated a substantial numerical trend favoring the control group, with a mean difference of −21.7 points (*p* = 0.090). This difference clearly exceeds the predefined MCID of 17.8 points, and Cohen’s *d* revealed a strong effect size (*d* = 0.64). Given this limited statistical power, the lack of significance despite a clinically meaningful difference strongly suggests a potential Type II error. This finding should be interpreted as a clinically relevant trend. This indicates that primary TKA patients might achieve superior high-demand sports function compared to the more complex combined rHTO+TKA procedure. This underscores that a lack of statistical significance must not be misinterpreted as proof of clinical similarity. While the descriptive parameters for the remaining scores overlap widely, the limited sample size prohibits a definitive conclusion on clinical comparability. The findings of this study are in line with the results of previous research by Radke and Radke [19]. The knee scores improved significantly to 76.00 (±21.19) [19]. Even though this was a single-stage TKA and femoral correction, follow-up studies by Arbeloa-Gutierrez et al. and Veltman et al. also showed good Knee Society Score (KSS) values postoperatively [20,31]. Madelaine et al. observed an increase in KSS following TKA and owHTO [18]. These findings support the feasibility and potential of achieving satisfactory functional outcomes using rHTO+TKA in selected patients [18,19,20,31].

Several biomechanical mechanisms explain why combined rHTO+TKA might lead to similar functional outcomes as standard TKA. Rather than performing massive asymmetric bone resections and radical soft tissue releases inside the joint, rHTO corrects the EAD directly at its apex. This restores a physiological joint line and normalizes the mechanical axis, optimizing load distribution. Consequently, collateral ligament integrity is fully preserved, allowing the exclusive use of standard CR implants. Retaining native ligament tension and joint kinematics ensures excellent stability and functional recovery with no significant differences compared to primary arthroplasty.

Clinically, single-stage rHTO+TKA offers an effective “one-shot” solution for symptomatic osteoarthritis secondary to failed HTO with valgus EAD, addressing both EAD and intra-articular arthritis while avoiding the burden of staged procedures. In this series, one of nine patients required revision for tibial plateau subsidence after osteotomy healing. In a small cohort, this single event represents a substantial 11.1% major complication rate. A detailed analysis of this specific case revealed that the female patient presented with underlying osteopenia and beginning osteoporosis, which reduced the structural bone quality and compromised mechanical stability. Revision surgery was successfully performed by removing the internal osteotomy plate and implanting a new cemented tibial component. This case underlines that despite the technical feasibility of a single-stage approach, diminished bone quality represents a critical risk factor, and ensuring rigid fixation remains paramount to prevent early mechanical failure. In line with current concepts of individualized alignment, this approach may offer advantages over standardized mechanical axis strategies, particularly in patients with valgus deformity and residual extra-articular malalignment following failed HTO.

### Limitations and Future Recommendations

A key strength of this study is the rigorous matched-pair design evaluating a highly complex and rare surgical procedure. However, this study has several limitations. Selecting a pristine primary TKA cohort without prior surgery as the control group represents a conceptual limitation. While this control group allowed us to benchmark the rHTO+TKA procedure against the clinical gold standard, comparing a complex salvage scenario to an unaltered primary anatomy sets a high baseline. Utilizing a control group consisting of TKA after prior HTO without concurrent rHTO would provide additional comparative value. The small sample size of nine matched pairs reduces statistical power, limits the ability to formally establish clinical equivalence based on MCID thresholds, and restricts generalizability to a broader patient population. The retrospective design also introduces potential selection bias, although the matched-pair methodology helped minimize confounding. Preoperative baseline values for the functional scores (OKS, LEFS, KOOS, FIPS) were not available due to the retrospective nature of the data, preventing the assessment of direct postoperative improvement or preoperative functional matching. In addition, matching by surgery date within a three-month window over a 10-year period may not be fully feasible and could introduce temporal bias related to changes in perioperative care over time. Furthermore, this single-center, single-surgeon experience ensures technical consistency but may reduce external validity in less specialized settings.

Regarding future research, the logical next step is to conduct larger multicenter prospective studies with long-term follow-up to validate these findings and to definitively evaluate potential clinical differences between rHTO+TKA and primary TKA. Additionally, exploring how patient-specific alignment strategies and robotic assistance can further optimize functional recovery in patients with severe EADs remains a critical area for future study.

## 5. Conclusions

This pilot study demonstrates the technical feasibility of single-stage rHTO and TKA, showing encouraging descriptive clinical and radiographic profiles in this small, highly specific cohort. Given the exploratory nature of this study, larger trials are required to definitively evaluate potential clinical differences.

## Figures and Tables

**Table 1:** Comparison of demographic and clinical characteristics between groups, presented as mean and standard deviation (SD), including statistical significance (*p*-values), each group *n* = 9 (rHTO: reversed high tibial osteotomy; TKA: total knee arthroplasty; BMI: body mass index; min = minutes; Hb: hemoglobin).

	Intervention Group (rHTO+TKA)	Control Group (TKA)	*p*-Value
Age (years) at operation	61.9 (±10.4)	61.3 (±9.9)	0.56
Female (%)	66.7	66.7	-
BMI (kg/m^2^)	28.4 (±4.9)	28.2 (±5.8)	0.79
ASA score	2	2	-
Smoker (%)	3 (33%)	2 (22%)	0.65
Hb preoperative (g/dL)	14.5 (±1.1)	14.2 (±0.7)	0.44
Hb postoperative (g/dL)	10.9 (±1.4)	11.6 (±1.0)	0.28
Surgery time (min)	117.0 (±24.2)	64.9 (±16.8)	<0.01

**Table 2:** Comparison of pre- and postoperative radiological parameters (in degrees) between the two groups; presented as mean and standard deviation (SD), including statistical significance (*p*-values), each group *n* = 9; negative values in aHKA indicate varus alignment (MPTA: medial proximal tibial angle; mLDFA: mechanical lateral distal femoral angle; aHKA: arithmetic hip–knee–ankle angle; JLO: joint line obliquity; rHTO: reversed high tibial osteotomy; TKA: total knee arthroplasty).

Angles (°)	Status	Intervention Group (rHTO+TKA)	Control Group (TKA)	*p*-Value
**MPTA**	preoperative	96.1 (±2.3)	87.1 (±2.6)	<0.001
	postoperative	90.2 (±3.0)	88.9 (±2.2)	0.368
**mLDFA**	preoperative	89.7 (±2.8)	89.0 (±2.8)	0.576
	postoperative	90.4 (±1.4)	91.8 (±2.7)	0.129
**aHKA**	preoperative	6.4 (±3.3)	−1.9 (±3.9)	0.001
	postoperative	−0.2 (±2.9)	−2.9 (±2.8)	0.104
**JLO**	preoperative	185.8 (±3.8)	176.1 (±3.8)	0.001
	postoperative	180.6 (±3.7)	180.7 (±4.1)	0.970

**Table 3:** Functional outcomes in TKA after rHTO compared with matched controls undergoing primary TKA; Values are presented as mean ± standard deviation (SD) and 90% confidence intervals (CIs) are reported (*n* = 9 pairs). Negative Δ-value indicates worse outcomes in the intervention group; positive Δ-values favor this group (LEFS: Lower Extremity Functional Scale; OKS: Oxford Knee Score; KOOS: Knee injury and Osteoarthritis Outcome Score, including subscales: Pain, Symptoms, ADL (Activities of Daily Living), Sports (Sports and Recreation), and QoL (Quality of Life); TAS: Tegner Activity Score (Δ: differences between pre- and postop); FIPS: Functional Index for Patients with Knee Disorders; TKA: total knee arthroplasty; rHTO: reversed high tibial osteotomy).

Score	Intervention Group (rHTO+TKA)	Control Group (TKA)	*p*-Value	90% CI of Δ	90% CI Within MCID
LEFS	77.4 (±22.6)	80.6 (±13.1)	0.736	[−20.2, 13.8]	No
OKS	39.3 (±10.9)	43.1 (±3.8)	0.314	[−10.3, 2.8]	No
KOOS Pain	81.2 (±26.0)	93.5 (±5.7)	0.171	[−27.6, 2.9]	No
KOOS Symptoms	70.2 (± 18.8)	73.8 (±18.0)	0.664	[−18.3, 11.2]	No
KOOS ADL	86.4 (±22.4)	94.0 (±8.3)	0.408	[−23.5, 8.5]	No
KOOS Sports	50.0 (±32.6)	71.7 (±25.9)	0.090	[−42.6, −0.8]	No
KOOS QoL	68.1 (±34.3)	77.8 (±16.9)	0.393	[−29.8, 10.3]	No
Δ TAS	−0.3 (±1.0)	0.4 (±1.3)	0.133	[−1.6, 0.1]	No
FIPS	2.3 (±1.1)	1.3 (±0.5)	0.053	[0.2, 1.8]	No

## Data Availability

The datasets used and analysed during the current study are available from the corresponding author on reasonable request.

## Abbreviations

aHKA	Arithmetic Hip–Knee–Ankle angle
ADL	Activities of Daily Living
ASA	American Society of Anesthesiologists
BMI	Body Mass Index
CI	Confidence Interval
CPAK	Coronal Plane Alignment of the Knee
CR	Cruciate-retaining
cwHTO	Closed Wedge High Tibial Osteotomy
EAD	Extra-articular Deformity
FIPS	Freiburg Index for Patient Satisfaction
Hb	Hemoglobin
HTO	High Tibial Osteotomy
JLO	Joint Line Obliquity
KOOS	Knee Osteoarthritis Outcome Score
LEFS	Lower Extremity Functional Score
mLDFA	Mechanical Lateral Distal Femoral Angle
MPTA	Medial Proximal Tibial Angle
OKS	Oxford Knee Score
owHTO	Open Wedge High Tibial Osteotomy
QoL	Quality of Life
rHTO	Reversed High Tibial Osteotomy
SD	Standard Deviation
TAS	Tegner Activity Score
TKA	Total Knee Arthroplasty
min	Minutes
g/dL	Grams per deciliter
kg/m^2^	Kilograms per square meter
°	Degrees
